# Fasted plasma asprosin concentrations are associated with menstrual cycle phase, oral contraceptive use and training status in healthy women

**DOI:** 10.1007/s00421-020-04570-8

**Published:** 2020-12-08

**Authors:** A. N. Leonard, A. L. Shill, A. E. Thackray, D. J. Stensel, Nicolette C. Bishop

**Affiliations:** 1 School of Sport, Exercise and Health Sciences, National Centre for Sport and Exercise Medicine, Loughborough University, Loughborough, UK; 2 English Institute of Sport, Loughborough, UK

**Keywords:** Female, Pre-menopausal, Birth control, Appetite hormone, Exercise

## Abstract

**Purpose:**

Asprosin, an orexigenic hormone that stimulates hepatic glucose release, is elevated in insulin resistance and associated with obesity. Plasma asprosin concentrations may also be related to female sex hormone levels; higher levels are reported in women with polycystic ovary syndrome (PCOS) but this may be related to peripheral insulin resistance also associated with PCOS. Clarification of female-specific factors influence on the plasma asprosin response is crucial for studies investigating asprosin. Therefore, this study determined the association of menstrual phase, oral contraceptive (OC) use (as a pharmacological influence on sex hormone levels) and training status (as a physiological influence on sex hormone levels) on plasma asprosin levels in pre-menopausal women.

**Methods:**

Fasting plasma asprosin, 17β-estradiol (E2) and progesterone, were assessed in 32 healthy untrained and trained women with regular menstrual cycles (non-OC; *n* = 8 untrained, *n* = 6 trained) or using OC (*n* = 10 untrained, *n* = 8 trained) during early follicular, late follicular and mid-luteal menstrual phases (or the time-period equivalent for OC users).

**Results:**

Asprosin was lower in OC (0.75 ± 0.38 ng mL^−1^) than non-OC users (1.00 ± 0.37 ng mL^−1^; *p* = 0.022). Across a cycle, asprosin was highest in the early follicular equivalent time-point in OC users (0.87 ± 0.37 ng mL^−1^) but highest in the mid-luteal phase in non-OC users (1.09 ± 0.40 ng mL^−1^). Asprosin concentrations varied more across a cycle in untrained than trained women, with higher concentrations in the early follicular phase compared to the late follicular and mid-luteal (training status-by-menstrual phase interaction *p* = 0.028).

**Conclusion:**

These findings highlight the importance of considering OC use, menstrual cycle phase and to a lesser extent training status when investigating circulating asprosin concentrations in females.

**Supplementary Information:**

The online version contains supplementary material available at 10.1007/s00421-020-04570-8.

## Introduction

Asprosin is a newly reported orexigenic protein hormone that is secreted from white adipose tissue and encoded by the final two exons of the FBN1 gene (Romere et al. 2016). FBN1 codes for the common extracellular matrix protein profibrillin 1 and it is the final terminus of this protein that makes up the hormone asprosin (Muthu and Reinhardt 2020). In a fasted state asprosin is elevated in the circulation, driving glucose release through a protein kinase A dependent mechanism (Romere et al. 2016).

The association between asprosin and appetite has been recently proposed; mice containing mutations affecting exon 65 of the FBN1 gene display hypophagia and extreme leanness compared to litter mates (Romere et al. 2016; Duerrschmid et al. 2017). In addition, these animals are protected from diabetes and obesity when exposed to dietary stress. Administration of recombinant asprosin rescues the hypophagia phenotype confirming the association with appetite. Clinical presentations in humans caused by mutations in FBN1 affecting asprosin are also associated with low subcutaneous adipose tissue and hypophagia, with significantly lower levels of circulating asprosin in those with mutations (Romere et al. 2016; Duerrschmid et al. 2017). In contrast, individuals who are overweight or have obesity have plasma asprosin concentrations which are up to fourfold above those of individuals with a healthy BMI (Ugur and Aydin 2019). This association of asprosin with body composition, presumably exerted through the effects on appetite, makes asprosin a target of significant interest in controlling energy balance and, therefore, disease in individuals with diabetes and obesity (Yuan et al. 2020).

Although the association with appetite has been demonstrated, elevated asprosin has been associated with negative consequences of diabetes, polycystic ovary syndrome (PCOS) and obesity, notably factors relating to insulin resistance, but a direct cause and effect relationship has yet to be robustly demonstrated in humans (Yuan et al. 2020). As research focusing on the role of asprosin in these conditions progresses, mounting data are being collected from women. Several of these studies have presented potentially contradictory results, but menstrual cycle phase and oral contraceptive (OC) use were not always controlled for (Li et al. 2018; Chang et al. 2019).

Metabolic disease is prevalent in both women and men, yet women are often excluded from research due to the cyclic changes in progesterone and oestrogen across a menstrual cycle (Sims and Heather 2018). Over a typical 28-day cycle, day 1 is the first day of menses marking the start of the follicular phase. The follicular phase is hallmarked by a steady rise in oestrogen which peaks at the end of the follicular phase (day 14; Reed and Carr 2000). The luteal phase is characterised by a steady rise in progesterone that peaks in the mid-luteal phase and gradually falls by the end of the luteal phase (Reed and Carr 2000). In contrast, users of OC have an almost completely blunted hormonal cycle due to inhibition of oestrogen and progesterone via exogenous hormones (Sims and Heather 2018). While a range of contraceptives are available, OC were reported by the United Nations (United Nation Department of Economic and Social Affairs Population Divison 2015) to be the most widely used form of contraceptive worldwide. Therefore, users form a large and distinct physiological group making it necessary to examine both OC users and non-users when examining female physiology (Sims and Heather 2018).

Female sex hormones are thought to play a role in metabolic processes such as insulin sensitivity and liver glucose release (Mauvais-Jarvis et al. 2013; Campbell and Febbraio 2002; Salpeter et al. 2006; Stubbins et al. 2012; Wilsterman et al. 2017). It is, therefore, essential to understand changes in circulating asprosin, a hormone thought to be involved in metabolism, over the menstrual cycle and in OC users so that reliable conclusions can be made in future research into asprosin.

While OC use presents a pharmacological influence on circulating sex hormone levels, physical training status provides a physiological influence on sex hormone levels. It is not uncommon for active women to have reduced oestrogen and progesterone release (De Souza 2003). With regard to asprosin, an 8 week aerobic running program in rats with type 1 diabetes elicited a lowering of hepatic asprosin concentrations (Ko et al. 2019). Studies in women are limited, yet one study reports increases in non-fasted circulating asprosin concentrations in healthy active women with normal menstrual cycles after a bout of exercise during the mid-follicular phase (Wiecek et al. 2018).

Therefore, the aim of this study was to determine the associations of menstrual phase, OC use (as a pharmacological influence on sex hormone levels) and training status (as a physiological influence on sex hormone levels) on plasma asprosin levels in pre-menopausal women. The findings of the study will help identify factors that need to be considered when assessing and interpreting asprosin data in females.

## Methods

### Participants

Healthy women (*n* = 32) with regular menstrual cycles or taking OC medication, for a minimum of three months, volunteered to participate in this observational study (Table 1). The women were untrained OC users (OC; *n* = 10), untrained non-OC users (non-OC; *n* = 8), trained oral contraceptive users (OC; *n* = 8) or trained non-oral contraceptive users (non-OC; *n* = 6). Trained was defined as participating in ≥ 3 h of structured purposeful exercise (e.g. team training or organised sessions) per week as determined by a self-reported training log. Trained and untrained women reported exercising 201–825 and 0–180 min per week, respectively. Trained women consisted of highly trained team sports athletes (*n* = 6), a recreationally trained team sports athlete (*n* = 1), highly trained endurance athletes (*n* = 4), a recreationally trained endurance athlete (*n* = 1), a highly trained middle-distance athlete (*n* = 1) and a highly trained power lifter (*n* = 1). Untrained women reported doing casual exercise such as going to the gym, attending exercise classes, running, walking, cycling and or no organised exercise at all.

**Table 1:** Participant characteristics in untrained and trained groups who use oral contraceptives (OC) or do not use OCs (non-OC). All values are presented as mean ± SD

	Untrained		Trained	
	OC (*n* = 10)	Non-OC (*n* = 8)	OC (*n* = 8)	Non-OC (*n* = 6)
Age (years)	24 ± 3	27 ± 5	24 ± 2	25 ± 5
Height (cm)	168.0 ± 7.5	169 ± 6.2	167.8 ± 5.1	166 ± 4.1
Body mass (kg)	63.1 ± 10.5	62.6 ± 7.6	62.1 ± 9.9	61.1 ± 7.3
BMI (kg/m^2^)	22.3 ± 2.8	21.9 ± 1.9	21.9 ± 2.7	22.2 ± 2.4
Exercising minutes (mean min week^−1^)	78 ± 52*	71 ± 70*	408 ± 204	382 ± 64
Menstrual cycle length (days)	28 ± 0	27 ± 2	28 ± 1	31 ± 6
Day ovulation detected	–	13 ± 2	–	16 ± 1

*Main effect of training status *p* ≤ 0.001

OC use was defined as using a monophasic OC for at least three months. A non-user (non-OC) was defined as no use of hormonal contraceptives for at least three months. Untrained OC reported taking: combined levonorgestrel (0.15 mg) and ethinylestradiol (0.03 mg; *n* = 6); combined norethisterone acetate (1.5 mg) and ethinylestradiol (0.03 mg; *n* = 1); combined desogestrel (0.15 mg) and ethinylestradiol (0.03 mg; *n* = 1); combined norgestimate (0.25 mg) and ethinylestradiol (0.035 mg; *n* = 1); or norethisterone (0.35 mg; *n* = 1). Trained OC reported taking: combined levonorgestrel (0.15 mg) and ethinylestradiol (0.03 mg; *n* = 3); combined norethisterone acetate (1.0 mg) and ethinylestradiol (0.03 mg; *n* = 2); combined desogestrel (0.15 mg) and ethinylestradiol (0.03 mg; *n* = 1); combined cyproterone acetate (2.0 mg) and ethinylestradiol (0.035 mg; *n* = 1); or combined dienogest (2.0 mg) and ethinylestradiol (0.03 mg; *n* = 1).

All participants completed a health-screen questionnaire and provided written informed consent to participate, after being informed of the rationale and protocol of the study. Further health-screen questionnaires and a willingness to participate form were completed at the beginning of each laboratory visit. The study protocol was approved by the local University Ethical Committee. In a preliminary visit a menstrual cycle questionnaire was completed to estimate cycle length and the following exclusion criteria were assessed: illnesses in the three weeks prior to laboratory testing; evidence of kidney disease or history of cardiovascular disease or metabolic disease; high blood pressure or dyslipidaemia; smokers or users of medical or illegal drugs that affect digestion, metabolism or inflammation; dieting or reported extreme dietary habits.

### Main visits

**Fig. 1:**
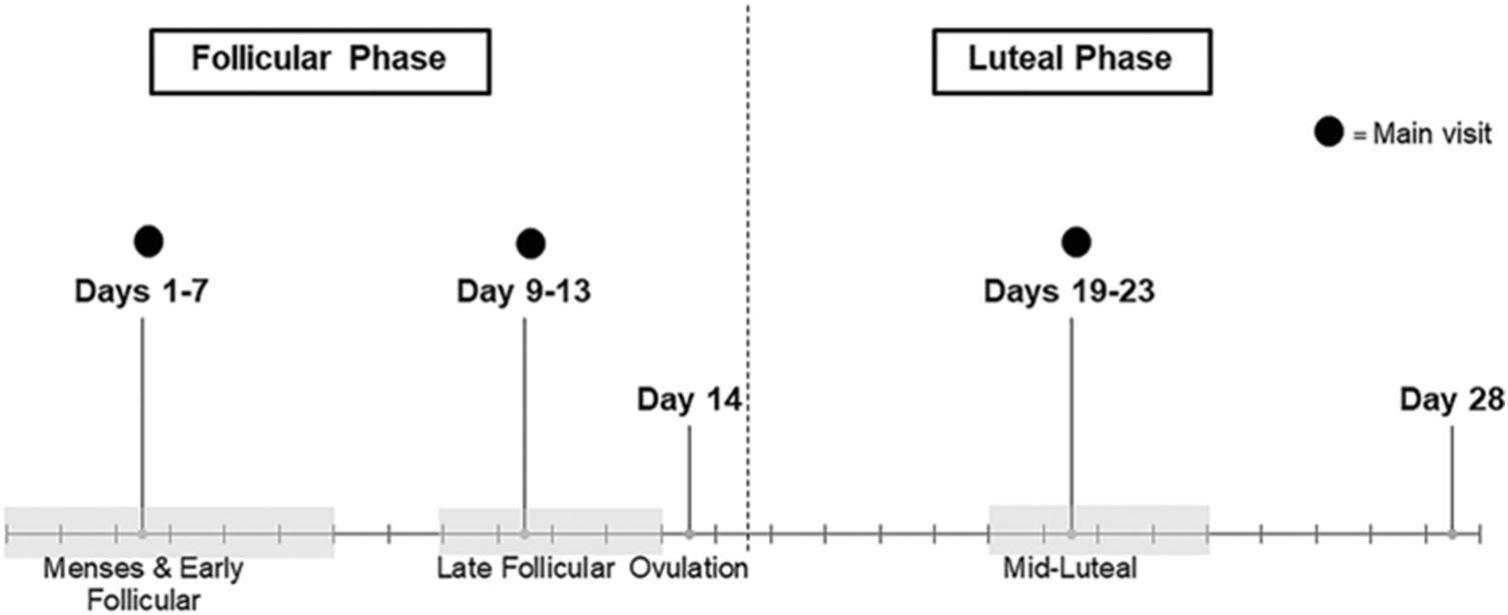
Menstrual cycle phases, or timepoint equivalents (for oral contraceptive users), when main visits occurred, based on a typical 28-day menstrual cycle, where fasted blood samples were taken and analysed for circulating concentrations of asprosin, 17β-estradiol and progesterone in healthy women

For non-OC users, the three laboratory visits were scheduled to capture the early follicular (first 6 days), late follicular (days 9–13) and mid-luteal (days 19–23) menstrual phases of a single menstrual cycle (Fig. 1). In OC users, these timepoints refer to the time period equivalent to the cycle phases in non-OC users. These were estimated based on a 28-day cycle with day 1 being the first day of menses or, for OC users, the first day without an OC dose resulting in a withdrawal bleed (Fig. 1). Participants were given commercially available ovulation midstream urine test sticks (Onestep, manufactured by AI DE Diagnostic Co. Ltd., China) to use for an eight-day period spanning the predicted ovulation day, commencing monitoring 3 days prior to the predicted ovulation day, following the manufacturer’s instructions. The results of each test were recorded on the participants’ diaries.

All laboratory visits began in the morning between 07:00 and 09:00 after an overnight fast of at least 10 h. Upon arrival, participants stature and body mass were measured followed by 15 min of seated rest before a venous blood sample was taken from an antecubital vein. After the final laboratory visit, participants contacted a member of the research team when menses next occurred to determine the total length of their cycle.

### Plasma analysis

Whole blood samples were collected into one 2.6 mL Lithium Heparin (16 IU heparin mL^−1^) and one 9 mL EDTA (1.6 mg mL^−1^; S-Monovette, Sarsdedt, Leicester UK) tube for female sex hormone and asprosin analyses, respectively. Blood was centrifuged at 4 °C for 10 min at 3500 rpm and plasma was extracted and frozen at − 80 °C for subsequent analysis.

Asprosin (ng mL^−1^) was determined in EDTA plasma using a commercially available enzyme-linked immunosorbent assay (ELISA), as per the manufacturer’s instructions (Abbexa, catalogue no. abx257694; Abbexa, Cambridge, UK). The within assay co-efficient of variation was 12.4%.

Heparinised plasma was assessed for 17β-estradiol (E2; pg mL^−1^) and progesterone (ng mL^−1^) using commercially available ELISAs, as per the manufacturer’s instructions (IBL International, Hamburg, Germany). The within assay co-efficient of variation for the duplicate analyses was 1.4% for E2 and 1.3% for progesterone.

### Statistical analyses

An a priori power calculation was conducted using G*Power 3.1.9.2 (Faul et al. 2007) based on the mean difference (and SD of the difference) in oestrogen concentrations between early and late follicular phases (given the rationale that asprosin would be influenced by menstrual phase and oestrogen levels). It was calculated that 24 participants (6 per group) would have > 95% power at the 0.05 level to detect a Cohen’s *d*_z_ of 1.77.

Using the statistical software SPSS v.24 (IBM Corporation) linear mixed models were used to examine differences in asprosin, E2 and progesterone concentration and body mass with fixed effects modelled of OC use (OC or non-OC), menstrual phase (early follicular, late follicular and mid-luteal) and training status (trained or untrained). Models were performed with repeated measures on the menstrual cycle variable. Where statistically significant main effects and interactions were identified, post-hoc analysis was performed using Fisher’s least significant difference.

Residuals were used to check for normality and distribution. Non-normally distributed data (E2 and progesterone) were logged, and models were run on the logged data. Absolute standardised effect sizes (ES) were calculated to supplement important findings using the descriptors outlined by Cohen (1988): < 0.2 trivial, 0.2–< 0.5 small, 0.5–< 0.8 moderate and ≥ 0.8 large. To retain clarity and interpretation, the data is displayed graphically to present the statistically significant main and interaction effects. This means that figures only depict significant group interactions (e.g. a fixed effect for the interaction between OC use and menstrual phase on plasma asprosin concentration does not include training status as a grouping variable when running the model and thus the figure would not include training status as an aspect for grouping means on the graph). However, individual data are displayed by OC use and training status in the Electronic Supplementary Material for transparency, but the means have been modelled and were not statistically significant when grouped as they are in the supplementary figures.

Pearson correlation coefficients were quantified to examine relationships between asprosin and sex hormones and any association with OC use. Thresholds of 0.1, 0.3 and 0.5 were used to define Pearson correlation coefficients as small, moderate and large, respectively (Cohen 1988). All data is reported as the mean ± standard deviation (SD). Statistical significance was accepted as *p* < 0.05. 95% confidence intervals (CI) for the mean absolute difference between groups were calculated.

## Results

### OC vs. non-OC

**Fig. 2:**
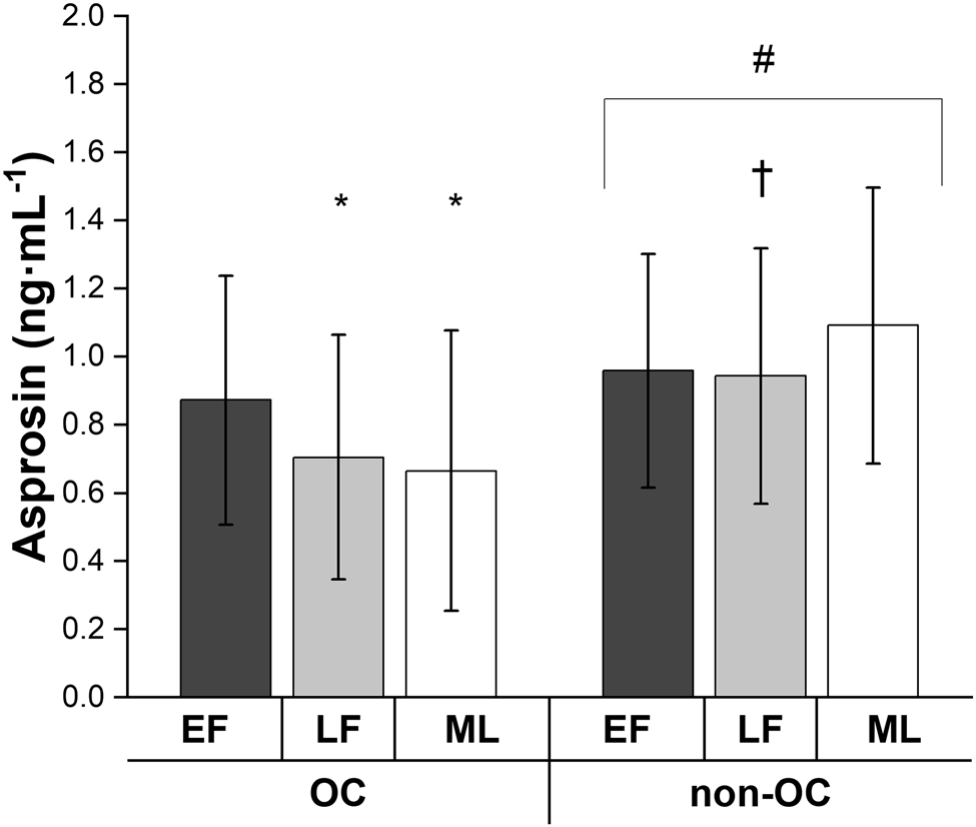
Fasted circulating plasma asprosin concentrations in oral contraceptive (OC) users and non-users (non-OC) in the early follicular (EF; first 6 days), late follicular (LF; days 9–13) and mid-luteal (ML; days 19–23) mensural cycle phases (or time point equivalent for OC users). ^†^Significantly lower than ML, within non-OC; *significantly lower than EF, within OC; ^#^significantly higher compared to OC; *p* < 0.05

Figure 2 displays asprosin concentrations across menstrual cycle phases in OC and non-OC users. Non-OC users had higher asprosin levels compared to OC users (main effect OC use *p* = 0.022; ES = 0.66 (moderate effect); 95% CI − 0.51, − 0.04 ng mL^−1^). Linear mixed models also showed an interaction between OC use and menstrual phase (*p* = 0.011). Post hoc analysis revealed that OC users had higher asprosin concentrations in early follicular compared to late follicular (*p* = 0.032; ES = 0.46 (small-to-moderate effect); 95% CI 0.01, 0.30 ng mL^−1^) and mid-luteal (*p* = 0.014; ES = 0.53 (moderate effect); 95% CI 0.04, 0.33 ng mL^−1^) phases whereas non-OC users had higher asprosin concentrations in mid-luteal compared to late follicular phase (*p* = 0.048; ES = 0.38 (small effect); 95% CI 0.001, 0.33 ng mL^−1^). For non-OC users, the 95% CI for the difference in asprosin concentration between the early follicular and the mid-luteal phase overlapped zero, and the standardised ES was small (*p* = 0.063; ES = 0.36; 95% CI − 0.01, 0.32 ng mL^−1^).

Asprosin positively correlated with progesterone (*p* = 0.019) and E2 (*p* = 0.009) among all 32 participants in the mid-luteal phase (Table 2). However, when the data were split by OC use the positive correlation of asprosin with progesterone was only seen in OC users (*p* = 0.001; Table 2).

**Table 2:** Pearson correlation (*r*) between plasma asprosin, oestrogen and progesterone in all women and women who use oral contraceptives (OC) or do not use OCs (non-OC) in the mid-luteal phase

	All women (*n* = 32)		
	Asprosin	Progesterone	Oestrogen
Asprosin	–	*r* = 0.418^a^	*r* = 0.462^b^
Progesterone		–	*r* = 0.642^b^

	OC (*n* = 18)			Non-OC (*n* = 14)		
	Asprosin	Progesterone	Oestrogen	Asprosin	Progesterone	Oestrogen
Asprosin	–	*r* = 0.735^b^	*r* = 0.383	–	*r* = − 0.008	*r* = 0.234
Progesterone		–	*r* = 0.523^a^		–	*r* = 0.517

Thresholds of 0.1, 0.3 and 0.5 were used to define Pearson correlation coefficients as small, moderate and large, respectively (Cohen 1988)

^a^Correlation is significant at the 0.05 level

^b^Correlation is significant at the 0.01 level

**Fig. 3:**
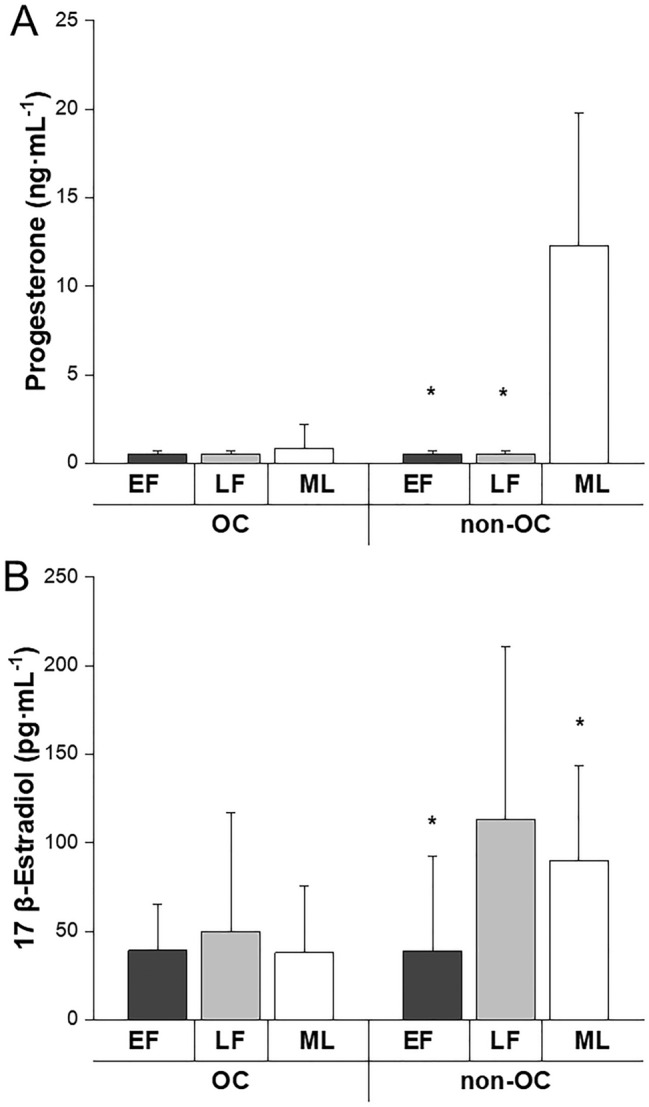
Fasted circulating plasma progesterone (**a**) and 17 β-Estradiol (**b**) concentrations in oral contraceptive (OC) users and non-users (non-OC) in the early follicular (EF; first 6 days), late follicular (LF; days 9–13) and mid-luteal (ML; days 19–23) mensural cycle phases (or time point equivalent for OC users). *Significantly (*p* ≤ 0.001) lower hormone values in EF and LF versus ML (panel **a**) and in EF and ML versus LF (panel **b**) within non-OC users

Figure 3 displays progesterone and E2 concentrations across menstrual cycle phases in OC and non-OC users. Linear mixed models also showed an interaction between OC use and menstrual phase for progesterone and E2 (*p* ≤ 0.046). Post hoc analysis revealed that progesterone and E2 values were similar across the menstrual cycle in OC users (*p* ≥ 0.142; ES ≤ 0.32 (small effect). In non-OC user’s, progesterone was higher in mid-luteal compared to early follicular (*p* < 0.0001; ES = 2.72 (large effect); 95% CI 9.22, 13.22 ng mL^−1^) and late follicular (*p* < 0.0001; ES = 2.69 (large effect); 95% CI 9.20, 13.21 ng mL^−1^) phases whereas E2 was lower in the early follicular compared to late follicular (*p* < 0.0001; ES = 1.21 (large effect) 95% CI − 106.17, − 30.86 pg mL^−1^) and mid-luteal (*p* < 0.0001; ES = 1.39 (large effect); 95% CI − 89.59, − 14.28 pg mL^−1^) phases.

Similar findings were apparent when the post-hoc analysis of the OC used by menstrual phase interactions were explored by comparing concentrations directly between groups at each cycle phase. Specifically, asprosin concentrations in the early follicular phase were not statistically different between OC (0.87 ± 0.37 ng mL^−1^) and non-OC (0.96 ± 0.34 ng mL^−1^) users (*p* = 0.379; ES = 0.24 (small effect); 95% CI − 0.38, 0.15). However, asprosin concentrations were higher in non-OC users than OC users in the late follicular (non-OC = 0.94 ± 0.37 ng mL^−1^; OC = 0.71 ± 0.36 ng mL^−1^; *p* = 0.05; ES = 0.65 (moderate effect); 95% CI 0.00, 0.53) and mid-luteal (non-OC = 1.09 ± 0.40 ng mL^−1^; OC = 0.67 ± 0.41 ng mL^−1^; *p* = 0.001; ES = 1.05 (large effect); 95% CI 0.19, 0.72) phases.

### Trained vs untrained

**Fig. 4:**
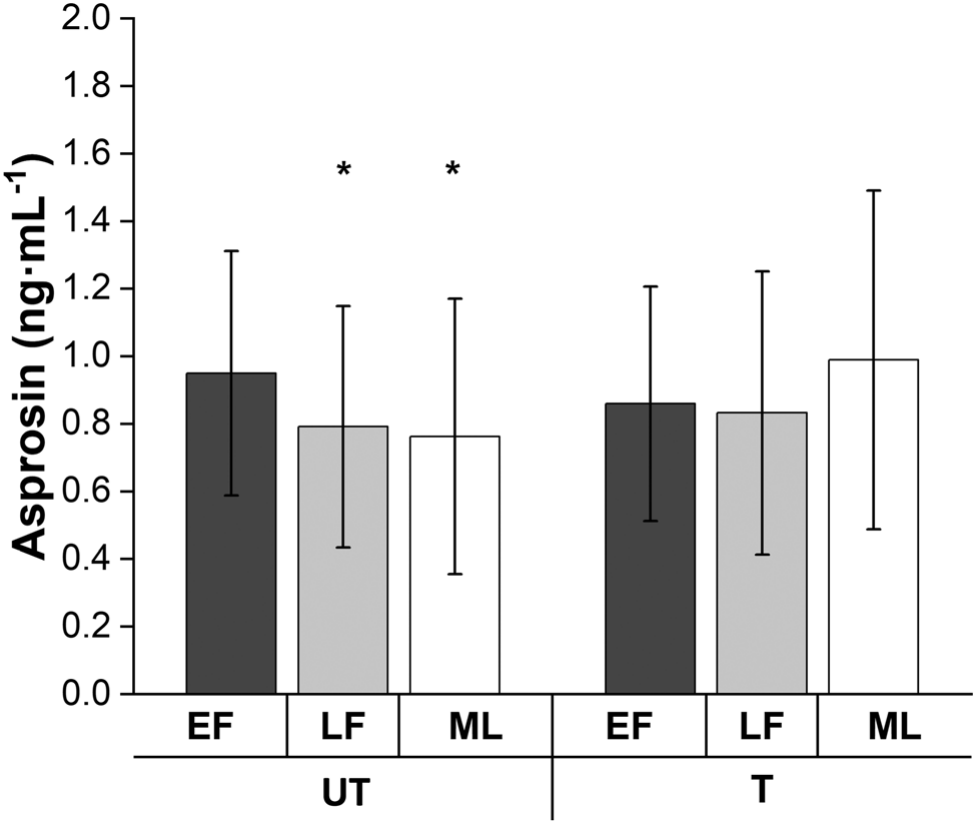
Fasted circulating plasma asprosin concentrations in untrained (UT) and trained (T) women in the early follicular (EF; first 6 days), late follicular (LF; days 9–13) and mid-luteal (ML; days 19–23) mensural cycle phases (or time point equivalent for OC users). *Significantly lower compared to EF, among R; *p* ≤ 0.045

Linear mixed models also showed an interaction between training status and menstrual phase on asprosin concentrations (*p* = 0.028). Post hoc analysis revealed that untrained, but not trained, women had significantly higher asprosin concentrations in the early follicular compared to late follicular (*p* = 0.045; ES = 0.44 (small-to-moderate effect); 95% CI 0.003, 0.29 ng mL^−1^) and mid-luteal (*p* = 0.023; ES = 0.49 (small-to-moderate effect); 95% CI 0.02, 0.31 ng mL^−1^; Fig. 4) phases. Circulating concentrations of E2 and progesterone were similar between trained and untrained women [main effect of training status *p* ≥ 0.526; ES ≤ 0.06 (trivial effect)].

### Three-way interaction between OC use, training status and menstrual phase

There were no statistically significant three-way interactions between OC use group, training status and menstrual cycle phase for asprosin, progesterone or E2 (*p* ≥ 0.245; see Figures in Electronic Supplementary Material). In the absence of existing asprosin data to perform an a priori power analysis for the interaction between OC use, training status and menstrual phase, a retrospective power calculation using G*Power (Faul et al. 2007) was conducted using the asprosin data in the present study. The three-way interaction between OC use, training status and menstrual cycle phase revealed a Cohen’s *f* of 0.08. Assuming an effect size (*f*) of 0.08, a post hoc power analysis revealed that the present analysis with 32 participants at the alpha level of 0.05 had 10% power for the three-way interaction between OC use, training status and menstrual cycle phase. Furthermore, it was estimated that a sample size of 380 (95 per group) participants would be required, for a three-way interaction, to detect the effect size (*f*) of 0.08 with an alpha level of 0.05 and 80% power.

## Discussion

The primary finding of the present study is that circulating fasted asprosin concentrations are associated with OC use and menstrual cycle phase in healthy young women. Importantly these findings suggest that OC use and menstrual phase should be controlled for when assessing and interpreting alterations in circulating asprosin levels. The present study reported that plasma asprosin: (1) is lower in OC users than in women with a normal menstrual cycle; (2) is highest in the early follicular equivalent time-point in OC users but highest in the mid-luteal phase in non-OC users, suggesting a potential phase-dependent change in plasma asprosin concentrations; and (3) demonstrated a phasic response across a menstrual cycle in untrained, but not highly trained, women.

Of note, OC users had lower plasma asprosin concentrations compared with non-OC users, and levels were highest in OC users in the early follicular phase, yet highest in the mid-luteal phase in non-OC users. In addition, asprosin concentrations were not different between OC users and non-OC users in the early follicular phase, menses, when OC users were not taking the pill. However, compared to non-OC, the OC users asprosin concentrations decreased across the timepoints, with the lowest asprosin concentrations in the mid-luteal timepoint where OC users have been taking the pill for the longest amount of time. In the present study ‘early follicular’ was defined as days 1–6 which captured the pill-free week in OC users. Since OC users’ asprosin concentrations were lower while taking OC (late follicular and mid-luteal) but higher in the pill-free week (early follicular) it is logical to infer that OC use is contributing to lower circulating plasma asprosin concentrations in women. OC users had 39% lower plasma asprosin compared to non-OC users in the mid-luteal phase. This difference is similar in magnitude to the difference in circulating plasma asprosin concentration in a nonfasted human to their fasted state (Romere et al. 2016) and emphasizes the need to control for OC use in the design of research studies assessing asprosin in women.

These findings suggest that OC use attenuates the natural phasic response of asprosin across the menstrual cycle. Amongst non-OC users, asprosin was highest in the mid-luteal compared to late follicular phase but asprosin mid-luteal concentrations were not significantly different from early follicular concentrations. This rise in asprosin concentration in the mid-luteal phase appears to be driven predominantly by trained women, although this is not statistically significant and, therefore, we cannot comment further on this trend (see electronic supplementary material Fig. 1). Across a typical menstrual cycle, the hallmark of the mid-luteal phase is a progesterone peak and plateau which occurs concomitantly with a smaller rise in oestrogen. However, the present study did not observe a correlation between asprosin and progesterone or E2 among non-OC users. This agrees with Chang et al (2019) who also did not see a correlation between E2 and asprosin in non-OC users. Furthermore, Li et al. (2018) also reported no correlation between asprosin and progesterone in women with PCOS, but did report a positive correlation between asprosin and E2 in normal weight (but not overweight) women with PCOS (Li et al. 2018). However, the latter study did not list OC use as an exclusion criterion for participants with PCOS. The present study did find a positive correlation between asprosin and progesterone among OC users only with the data was split by OC use and all participants in the present study were of normal weight. The inclusion of both OC users and non-OC users in the study by Li et al. (2018) could explain the disparity in findings and highlights the need to control for OC use when examining circulating asprosin concentrations in women.

Studies have found a positive correlation between testosterone and asprosin in women with PCOS (Li et al. 2018; Chang et al. 2019). In addition, Chang et al. (2019) reported a correlation between asprosin and follicle-stimulating hormone. Considering these studies involved women with diagnosed PCOS, a condition known to effect androgens, this could account for their findings (Goodarzi et al. 2011). It is possible that asprosin interacts with other sex hormones not measured in the present study, which may elevate asprosin concentrations in the mid-luteal phase. Therefore, further research is needed into circulating asprosin concentrations and sex hormones such as testosterone and follicle-stimulating hormone. In the meantime, it is prudent to account for menstrual cycle phase in the design of future studies until it is clear how asprosin is affected by sex hormones.

The present study demonstrated higher asprosin concentrations only amongst untrained women in the early follicular compared to late follicular and mid-luteal phases. The increased asprosin concentration in the early follicular phase appears to be driven predominantly by OC users, although this is not statistically significant and, therefore, we cannot comment further on this trend (see electronic supplementary material Fig. 1). This mirrors the increase in asprosin during the pill-free week observed the OC users, as the timing of the early follicular phase would coincide with the pill free week. In contrast, asprosin concentrations did not show a phasic response in trained women, irrespective of OC use, suggesting that training status does influence asprosin concentrations. As previously mentioned, testosterone and follicular stimulating hormone have been reported to correlate with asprosin concentrations in studies with less control on OC use and menstrual phase in females with PCOS (Li et al. 2018; Chang et al. 2019). Since it is possible that some sex-related homones are correlated with asprosin it could be that hormones not measured in the present investigation were suppressed or increased in our trained women which prevented the natural cycling of asprosin concentrations to occur (Cho et al. 2017). Our findings also suggest training status may need to be considered when measuring novel biomarkers or variables known to be influenced by the menstrual cycle due to potential luteal phase anomalies that may affect results.

The findings of the present study are preliminary and require confirmation in future research. A priori power calculations based on meaningful changes in oestrogen along with our inclusion of effect sizes allow our results to be interpreted with more confidence. Although, to examine a specific three-way interaction between OC use, training status and menstrual cycle phase (see Figures in Electronic Supplementary Material) a future study with larger sample size is required, as demonstrated by our retrospective power analysis. That said, our calculations suggest a sample size of almost 400 women which may limit the feasibility of conducting such a study. However, given the difference in asprosin between OC user groups and the pattern of change in concentrations across the menstrual cycle was small-to-moderate in magnitude, it may be prudent to consider OC use when investigating asprosin responses in women. It should also be acknowledged that various types of monophasic OCs were used by women in the present study, all of which could exert different magnitude of effects on asprosin.

In conclusion, the present study demonstrated markedly lower plasma asprosin concentrations in OC users compared to women who are not taking OC and have a normal menstrual cycle. Furthermore, asprosin concentrations appear to be influenced by menstrual cycle phase in non-OC users and demonstrated greater variation across a cycle in untrained than trained women. Therefore, OC use, menstrual cycle phase and training status should be considered in the design of future studies assessing and interpreting asprosin data in females.

## Supplementary Information

Below is the link to the electronic supplementary material.

Supplementary file1 (EPS 31194 KB)

Supplementary file2 (EPS 71428 KB)

Supplementary file3 (DOCX 22 KB)

## Data Availability

The datasets generated during and/or analysed during the current study are available from the corresponding author on reasonable request. Relevant individual data are also included in the Electronic Supplementary Material.

## Abbreviations

E2: 17β-Estradiol
EF: Early follicular
ELISA: Enzyme-linked immunosorbent assay
ES: Effect size
LF: Late follicular
ML: Mid-luteal
OC: Oral contraceptive
PCOS: Polycystic ovary syndrome
SD: Standard deviation
T: Trained
UT: Untrained
