# Supplementary material for: Fasted plasma asprosin concentrations are associated with menstrual cycle phase, oral contraceptive use and training status in healthy women
Source: Eur J Appl Physiol. 2020 Dec 8;121(3):793–801. doi: 10.1007/s00421-020-04570-8 (PMC7892699; doi:10.1007/s00421-020-04570-8)
Supplement: Supplementary file 3 — Supplementary file3 (DOCX 22 KB) [file 421_2020_4570_MOESM3_ESM.docx]

**Electronic Supplementary Material Captions**

**ESM Fig. 1** Plasma asprosin concentrations in recreationally active and trained oral contraceptive users (□) and non-oral contraceptive users (○) with dotted lines connecting individual data points. The bars represent means and error bars represent the standard deviation

**ESM Fig. 2** Plasma progesterone (**A**) and 17 β-Estradiol (**B**) concentrations in recreationally active and trained oral contraceptive users (□) and non-oral contraceptive users (○) with dotted lines connecting individual data points. Bars represent geometric means and error bars represent the 95% CI
